# Supplementary material for: Structural Insights into the Marine Alkaloid Discorhabdin G as a Scaffold towards New Acetylcholinesterase Inhibitors
Source: Mar Drugs. 2024 Apr 12;22(4):173. doi: 10.3390/md22040173 (PMC11051419; doi:10.3390/md22040173)
Supplement: Supplementary file 1 [file marinedrugs-22-00173-s001.zip › marinedrugs-2950221-supplementary.pdf]

Supplementary Materials

Structural Insights into the Marine Alkaloid Discorhabdin G as a Scaffold for New Acetylcholinesterase Inhibitors

Andrea Defant [1,*], Giacomo Carloni [1,2], Nicole Innocenti [1], Tomaž Trobec [3], Robert Frangež [3], Kristina Sepčić [4] and Ines Mancini [1,*]

[1] Laboratory of Bioorganic Chemistry, Department of Physics, University of Trento, Via Sommarive 14, 38123 Trento, Italy

[2] Present address: Unité de Microbiologie Structurale, Institut Pasteur, CNRS, Université Paris Cité, Paris 75015, France; giacomo.carloni@pasteur.fr

[3] Institute of Preclinical Sciences, Veterinary Faculty, University of Ljubljana, Gerbičeva 60, 1000 Ljubljana, Slovenia; tomaz.trobec@vf.uni-lj.si; robert.frangez@vf.uni-lj.si

[4] Department of Biology, Biotechnical Faculty, University of Ljubljana, Jamnikarjeva 101, 1000 Ljubljana, Slovenia; kristina.sepcic@bf.uni-lj.si

* Correspondence: ines.mancini@unitn.it (I.M.); andrea.defant@ex-staff.unitn.it (A.D.)

Contents

Table S1. Distribution (%) among neutral structure and protonated forms of the compounds **2** and **3** as a function of pH values, as evaluated by MarvinSketch software.

Figure S1. Druglikeness prediction for (a)discorhabdin G (**1**), (b) compound **2** and (c) compound **3** by Molsoft L.L.C.

Figure S2. Purity evaluation of **3**, **7**, and **8** subjected to bioassays by HPLC analysis (Phenomenex Gemini 5μm C18 110A column, in gradient conditions (CH_3CN t_0 30%, $t_{8\ min}$ 80%, and $t_{22\ min}$ 80%), flow 1 mL/min, λ 254nm.

Figure S3. Inhibition of electric eel acetylcholinesterase (eeAChE, ●), human recombinant acetylcholinesterase (hAChE, ●) or horse serum butyrylcholinesterase (BChE, ●) by increasing concentrations of compounds **3** (**A**), **7** (**B**) and **8** (**C**). The activity was monitored as described in the Chapter 3.2.

Figure S4. Determination of type of inhibition and inhibition constants (Ki) for (a) compound **3** and (b) compound **7** towards electric eel acetylcholinesterase (eeAChE), human recombinant acetylcholinesterase (hAChE), and horse serum butyrylcholinesterase (BChE) by Dixon plot analysis. Substrate (acetylthiocholine chloride) concentrations: 0.125 mM (▲), 0.25 mM (●), 0.5 mM (■).

Figure S5. Two-dimensional views of the interactions, by AutoDock Vina calculation, between *T. californica* AChE (6G1V) and **7** and **8**.

Figure S6. ^{1}H NMR spectrum (400 MHz, in $CDCl_3$) of compound **3** as acetate salt, and ^{13}CNMR spectrum (100 MHz, in $CDCl_3$) of neutral **3**.

Figure S7. ^{1}H and ^{13}CNMR spectra in $CDCl_3$, at 400 MHz and 100 MHz respectively of compound **7**.

Figure S8. ^{1}H and ^{13}CNMR spectra in $CDCl_3$, at 400 MHz and 100 MHz respectively of compound **8**.

Table S1. Percentage distribution among neutral structure A and protonated forms of the compounds **2** and **3** as a function of pH values, as evaluated by MarvinSketch software.

		**Compound 2**		
pH	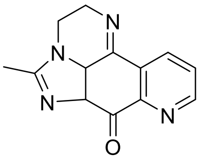	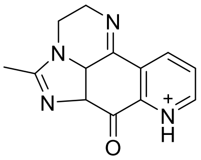	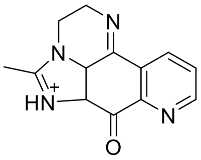	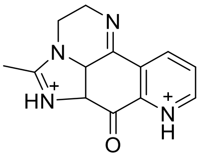
4.00	52.499	0.125	47.293	0.078
5.00	91.714	0.022	8.262	0.001
7.40	99.964	0.000	0.036	0.000
9.00	99.999	0.000	0.001	0.000
		**Compound 3**		
pH	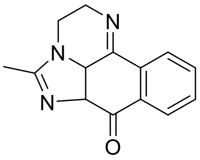	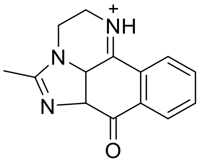	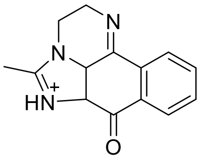	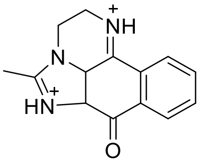
4.00	46.885	1.733	50.841	0.541
5.00	89.908	0.332	9.749	0.010
7.40	99.955	0.001	0.043	0.000
9.00	99.999	0.000	0.001	0.000

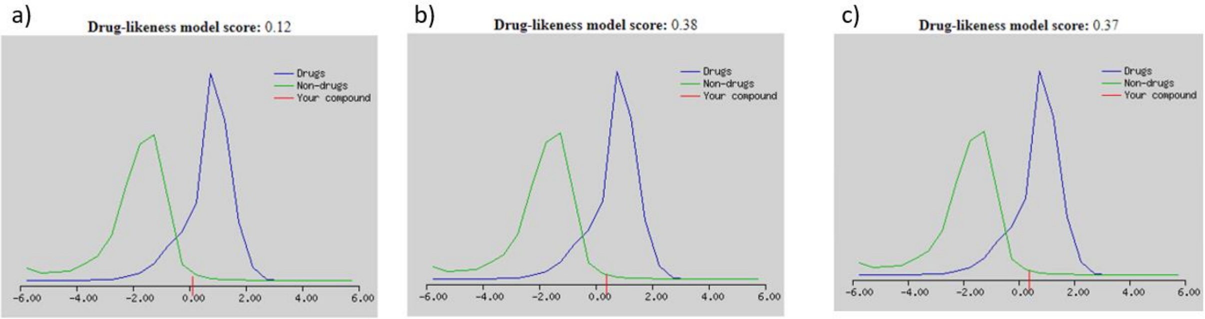

Figure S1. Druglikeness prediction for discorhabdin G(**1**), (a); compounds **2** (b) and **3** (c) by Molsoft L.L.C.

Compound 3

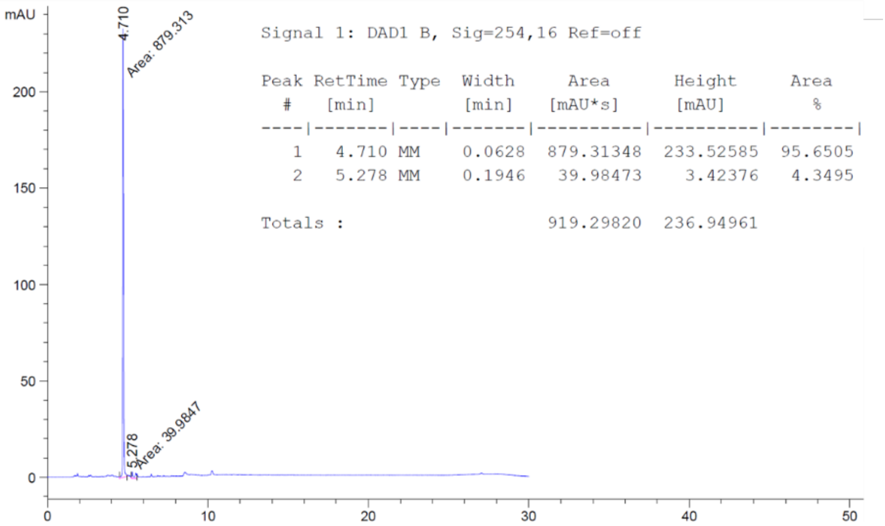

Signal 1: DAD1 B, Sig=254,16 Ref=off

Peak #	RetTime [min]	Type	Width [min]	Area [mAU*s]	Height [mAU]	Area %
1	4.710	MM	0.0628	879.31348	233.52585	95.6505
2	5.278	MM	0.1946	39.98473	3.42376	4.3495
Totals :				919.29820	236.94961	

Compound **7**

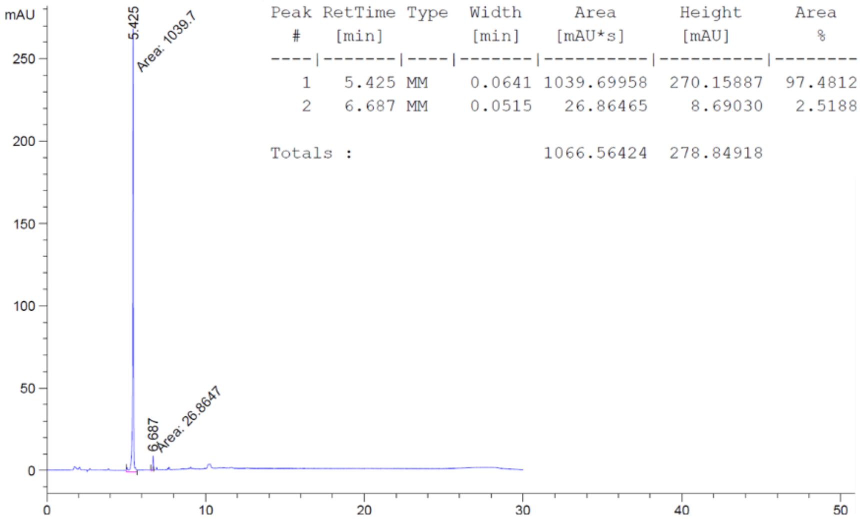

Peak #	RetTime [min]	Type	Width [min]	Area [mAU*s]	Height [mAU]	Area %
1	5.425	MM	0.0641	1039.69958	270.15887	97.4812
2	6.687	MM	0.0515	26.86465	8.69030	2.5188
Totals :				1066.56424	278.84918	

Compound **8**

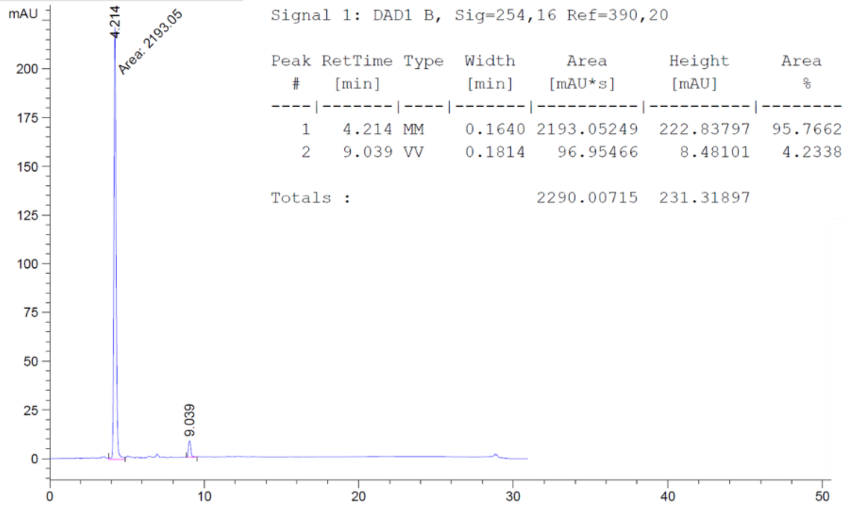

Signal 1: DAD1 B, Sig=254,16 Ref=390,20

Peak #	RetTime [min]	Type	Width [min]	Area [mAU*s]	Height [mAU]	Area %
1	4.214	MM	0.1640	2193.05249	222.83797	95.7662
2	9.039	VV	0.1814	96.95466	8.48101	4.2338
Totals :				2290.00715	231.31897	

Figure S2. Purity evaluation of **3**, **7**, and **8** subjected to bioassays by HPLC analysis subjected to bioassays (Phenomenex Gemini 5μm C18 110A column, in gradient conditions (CH_3CN t_0 30%, $t_{8\ min}$ 80%, and $t_{22\ min}$ 80%), flow 1 mL/min, λ 254nm.

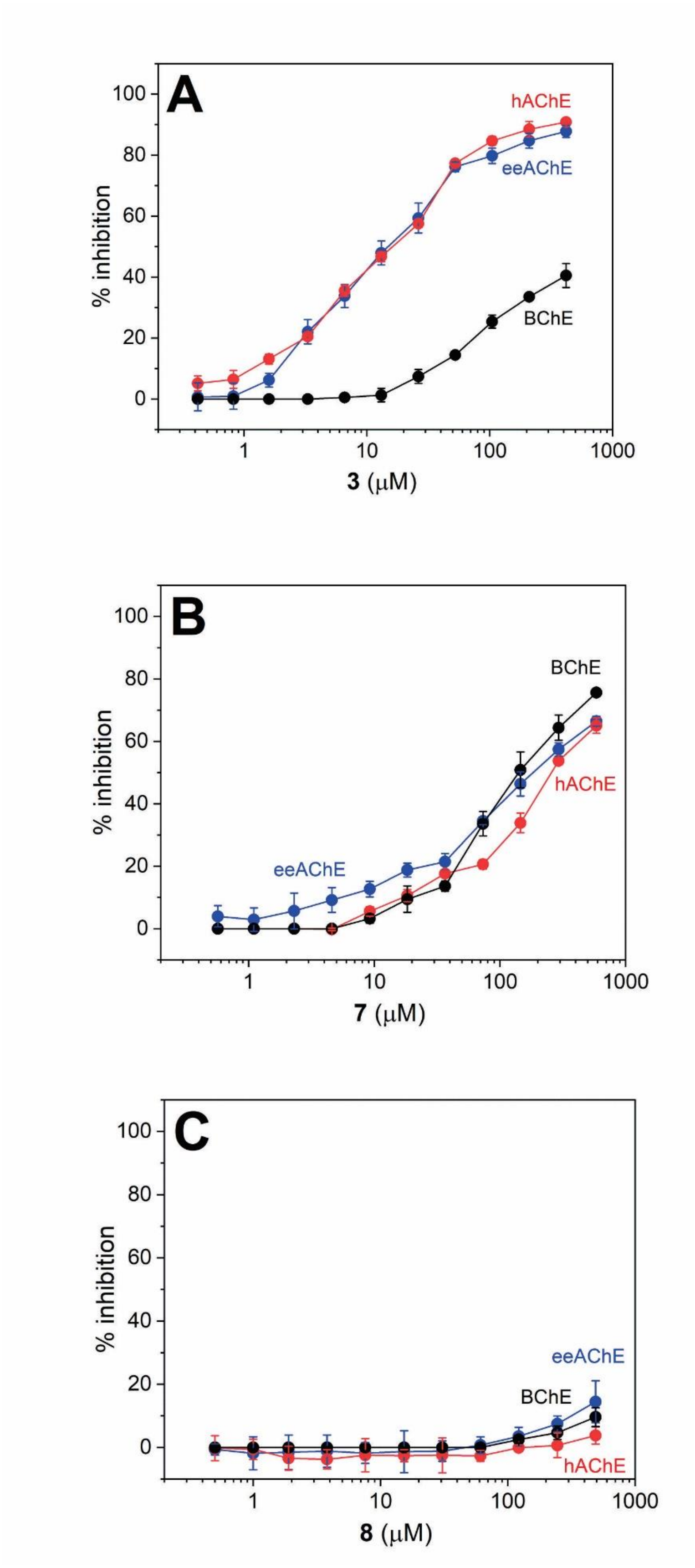

Figure S3. Inhibition of electric eel acetylcholinesterase (eeAChE, ●), human recombinant acetylcholinesterase (hAChE, ●) or horse serum butyrylcholinesterase (BChE, ●) by increasing concentrations of compounds **3** (**A**), **7** (**B**) and **8** (**C**). The activity was monitored as described in the Chapter 3.2.

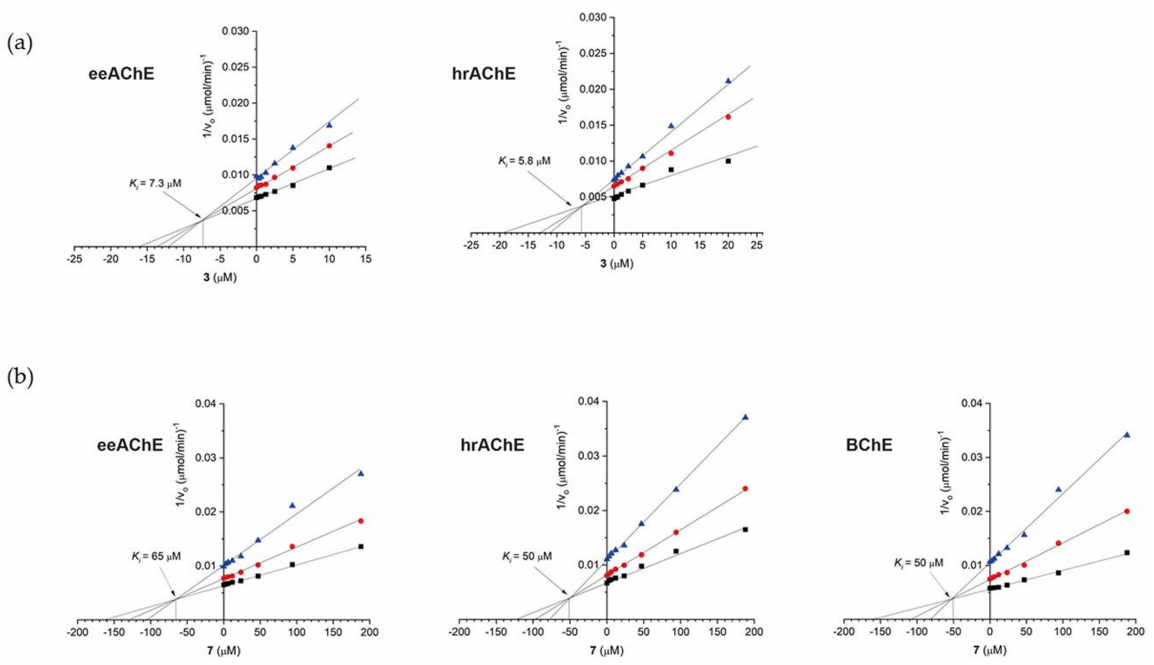

Figure S4. Determination of type of inhibition and inhibition constants (Ki) for (a) compound **3** and (b) compound **7** towards electric eel acetylcholinesterase (eeAChE), human recombinant acetylcholinesterase (hAChE), and horse serum butyrylcholinesterase (BChE) by Dixon plot analysis. Substrate (acetylthiocholine chloride) concentrations: 0.125 mM (▲), 0.25 mM (●), 0.5 mM (■).

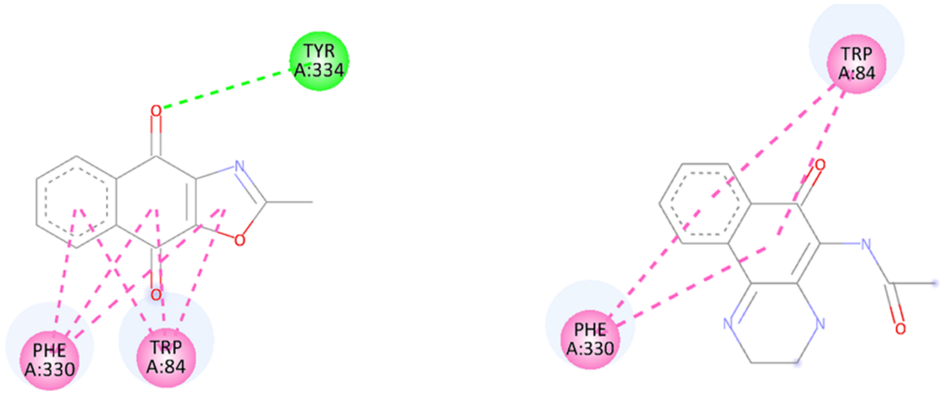

7 8

Figure S5. Two-dimensional views of the interactions, by AutoDock Vina calculation, between *T. californica* AChE (6G1V) and compounds **7** and **8**.

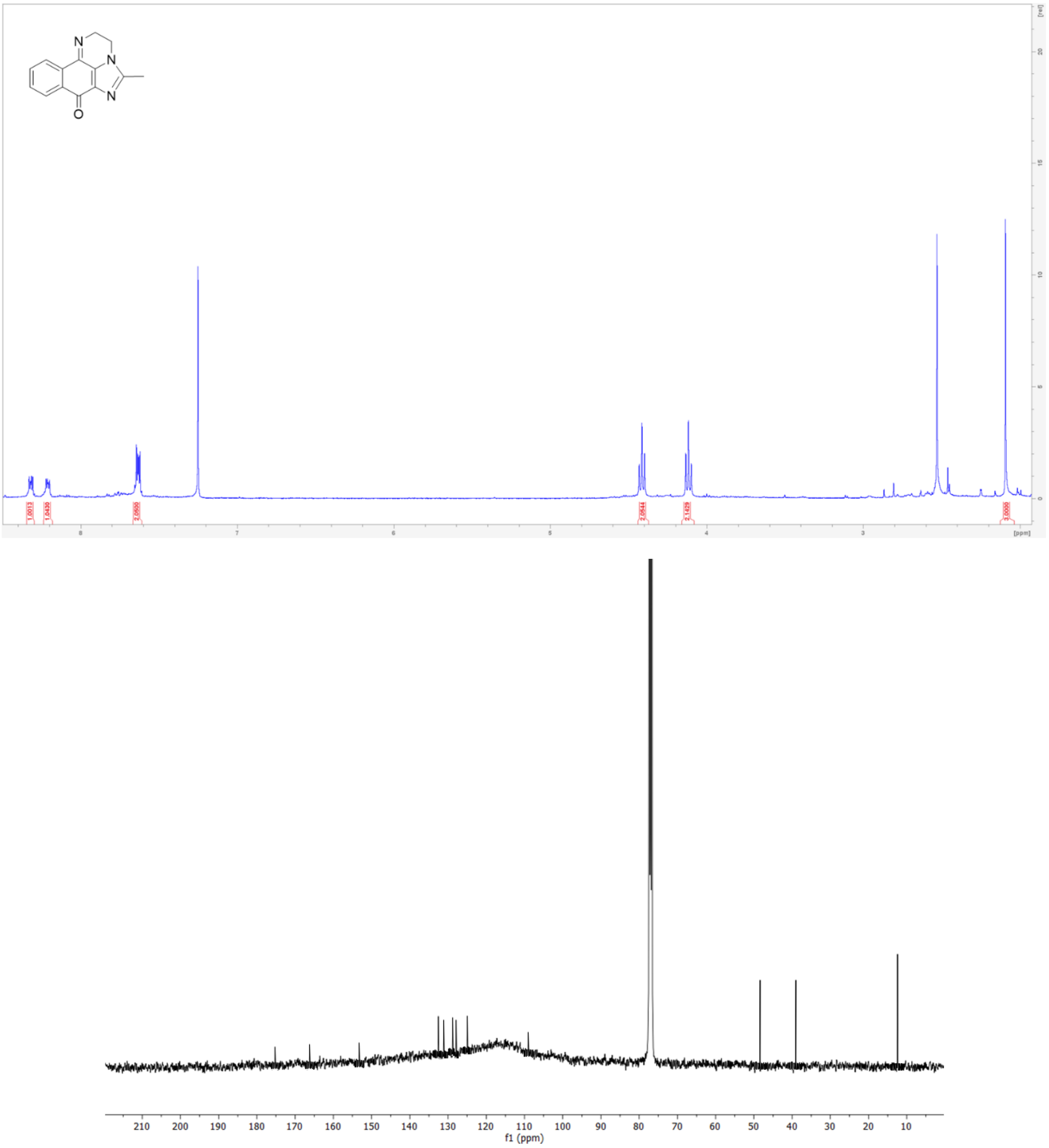

Figure S6. ^{1}H NMR spectrum (400 MHz, in $CDCl_3$) of compound **3** as acetate salt, and ^{13}CNMR spectrum (100 MHz, in $CDCl_3$) of neutral **3**.

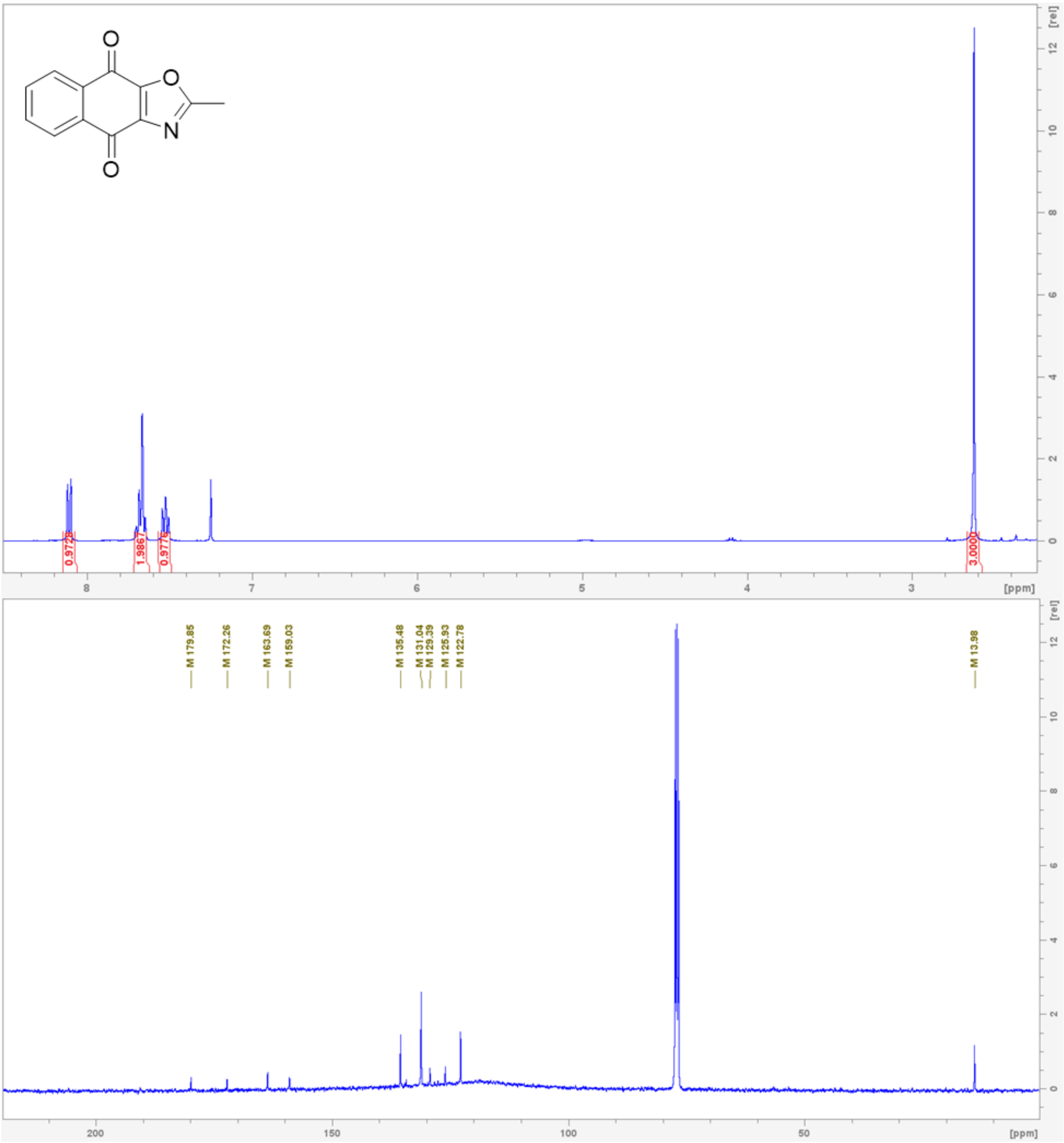

Figure S7. ^{1}H and ^{13}CNMR spectra in $CDCl_3$, at 400 MHz and 100 MHz respectively of compound **7**.

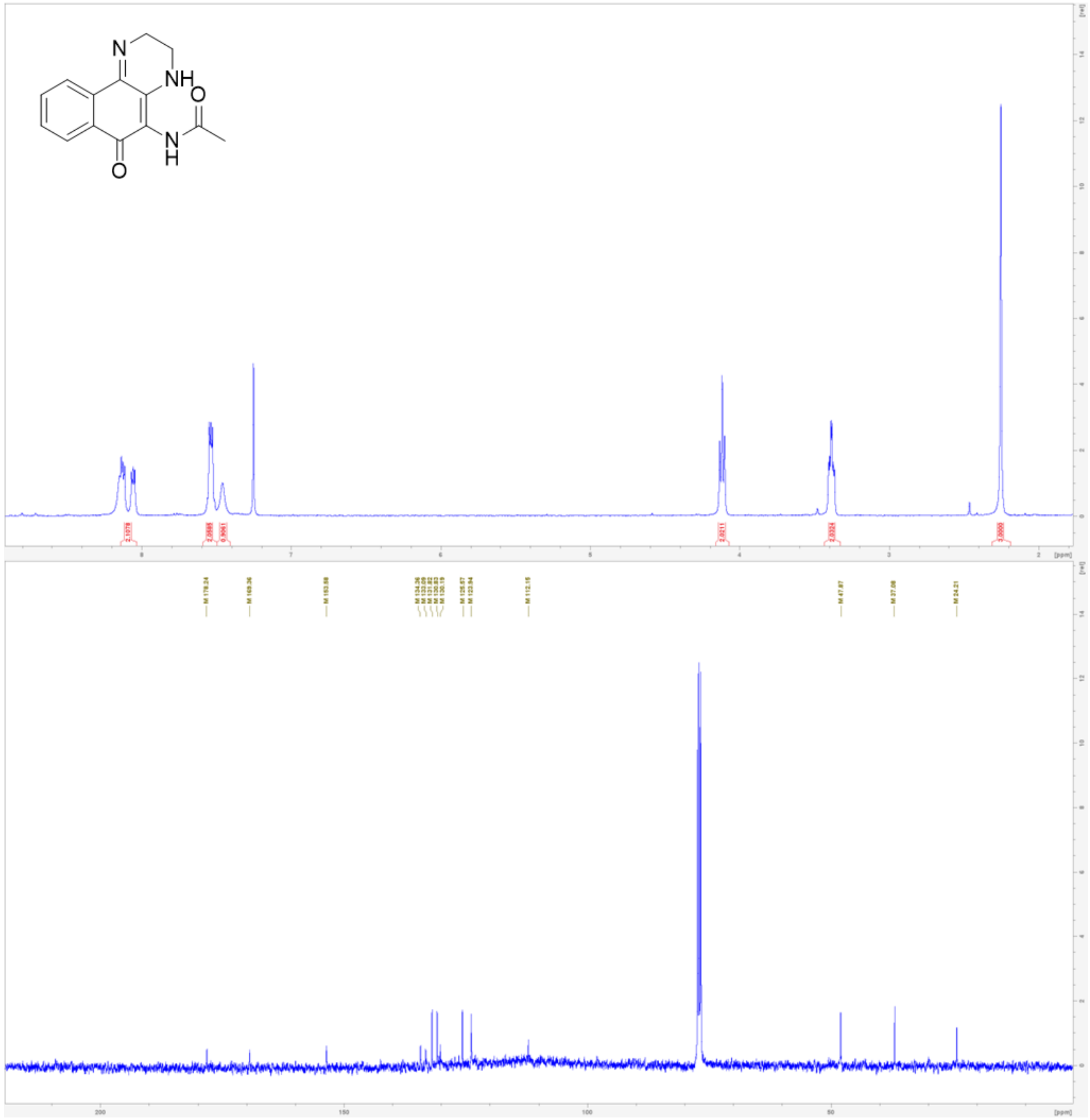

Figure S8. ^{1}H and ^{13}CNMR spectra in $CDCl_3$, at 400 MHz and 100 MHz respectively of compound **8**.
